# The Effect of Acute Supplementation of Branched Chain Amino Acids on Serum Metabolites During Endurance Exercise in Healthy Young Males: An Integrative Metabolomics and Correlation Analysis Based on a Randomized Crossover Study

**DOI:** 10.3390/metabo16010041

**Published:** 2026-01-02

**Authors:** Xinxin Zhang, Xintang Wang, Chenglin Luan, Yizhang Wang, Junxi Li, Wei Shan, Zhen Ni, Chunyan Xu, Lijing Gong

**Affiliations:** 1 China Institute of Sport and Health Science, Beijing Sport University, Beijing 100084, China; 2 Key Laboratory for Performance Training & Recovery of General Administration of Sport, Beijing 100084, China; 3 Key Laboratory of Exercise and Physical Fitness of Ministry of Education, Beijing Sport University, Beijing 100084, China; 4 School of Sport Science, Beijing Sport University, Beijing 100084, China; 5 Beijing Higher School Engineering Research Center of Sport Nutrition, Beijing Sport University, Beijing 100084, China

**Keywords:** branched-chain amino acids, untargeted metabolomics, sports nutrition, energy metabolism, dual regulation

## Abstract

Background: Branched-chain amino acids (BCAAs) are popular as sports supplements due to their ability to enhance performance and recovery. However, the full spectrum of metabolic alterations triggered by acute supplementation with BCAAs in conjunction with exercise remains incompletely understood. Methods: A randomized crossover trial was conducted in 8 healthy active young males, who received either BCAA or placebo supplementation for three consecutive days prior to a high-intensity cycling test. Plasma samples were collected pre- and post-exercise and analyzed by ultra-high-performance liquid chromatography–quadrupole time-of-flight mass spectrometry, followed by correlation and enrichment analyses. Results: Acute BCAA supplementation was significantly associated with enhanced fat oxidation and attenuated post-exercise increases in plasma ammonia, creatine kinase, and lactate dehydrogenase, suggesting the potential improvements in energy supply and membrane stability. Metabolomics analysis identified differential metabolites primarily involved in lipid, amino acid, and glucose metabolism. Pathway enrichment revealed coordinated regulation of fatty acid oxidation (FAO) and tryptophan-related pathways. Correlation analysis further showed that changes in metabolite profiles were strongly associated with biochemical outcomes, particularly linking enhanced fat oxidation and ammonia clearance with BCAA intake. Conclusions: Short-term BCAA supplementation could enhance FAO and membrane stability via coordinated regulation of lipid and amino acid metabolism post exercise, supporting its potential role as a precision nutrition strategy.

## 1. Introduction

Vigorous exercise can induce obvious metabolic stress, depleting energy stores and causing muscle protein damage, which underscores the importance of targeted nutritional strategies for optimal recovery [1]. Among these strategies, branched-chain amino acids (BCAAs)—leucine, isoleucine, and valine—are widely used for their dual role as signaling molecules for protein synthesis and substrates for energy production [2,3,4]. BCAAs are also deeply involved in key metabolic processes such as lipid oxidation and the tricarboxylic acid (TCA) cycle [2,5,6]. For example, BCAAs supplementation can enhance carnitine-mediated fatty acid transport, thereby improving exercise efficiency and reducing post-exercise fatigue [5]. Beyond their anabolic function, BCAAs may serve as alternative fuel sources during prolonged or glycogen-depleting exercise. However, this can also lead to the production of BCAA-derived metabolites and potentially contribute to a state known as “anabolic resistance,” characterized by diminished muscle protein synthesis and a reduced adaptive response to training, often linked with chronic fatigue, inflammation, or dysregulated protein turnover.

Additionally, BCAAs have been associated with the attenuation of both central and peripheral fatigue [5,7,8]. During prolonged strenuous exercise, the elevated brain tryptophan levels can promote serotonin (5-HT) synthesis, which is linked to fatigue perception. BCAAs compete with tryptophan for transport across the blood–brain barrier, and then lower the production of 5-HT and potentially delay central fatigue [5]. Furthermore, BCAA intake can attenuate metabolic acidosis and the accumulation of ammonia, maintain the levels of blood glucose and free fatty acid, and reduce increases in muscle damage markers such as creatine kinase (CK) and lactate dehydrogenase (LDH), collectively supporting more efficient recovery [9].

Meanwhile, the efficacy of BCAAs supplementation appears to be modulated by several factors, including exercise intensity, modality, and individual nutritional status [10]. BCAA supplementation after exercise is primarily recognized for alleviating delayed-onset muscle soreness (DOMS) and promoting muscle repair. Systematic reviews indicate that in trained individuals, continuous intake (≤255 mg/kg/day) of BCAAs after moderate muscle damage significantly reduces soreness within 24–72 h and accelerates functional recovery [11]. Notably, the efficacy of BCAA supplementation is modulated by exercise intensity, with more pronounced effects observed during prolonged or high-intensity exertion, whereas benefits are less consistent in low-intensity scenarios. These variable outcomes suggest that BCAAs exert multifaceted, context-dependent influences on energy expenditure and recovery processes. While previous studies have often focused on isolated metabolic parameters—such as respiratory exchange ratio (RER), β-hydroxybutyrate, or ammonia—this targeted approach may overlook the broader, coordinated metabolic adaptations induced by BCAA supplementation following exercise.

In this context, non-targeted metabolomics offers a powerful tool for capturing global changes in metabolites such as amino acids, lipids, and other metabolites, thereby providing a comprehensive profile of the metabolomic response to both exercise and nutritional interventions [9,11]. Previous applications of this approach have successfully revealed dynamic shifts in fatty acids, carnitine derivatives, and TCA cycle intermediates in response to exercise, reflecting underlying substrate utilization and energy metabolism transitions [12,13]. Although numerous studies have examined the role of BCAAs in regulating exercise metabolism, few have systematically employed metabolomics to elucidate how BCAAs coordinately regulate fatty acid oxidation (FAO), amino acid metabolism, and mitochondrial function in an integrated manner [14].

Therefore, the objective of this study was to investigate the effects of acute BCAA supplementation on pre- or post-exercise energy metabolism in young males, using untargeted plasma metabolomics, aiming to elucidate the mechanisms by which BCAAs promote energy replenishment and muscle recovery. We hypothesized that BCAA supplementation would enhance post-exercise recovery by coordinately modulating FAO, amino acid metabolism, and mitochondrial function, as reflected in the global metabolomic profile. These findings may contribute to the development of precision nutrition strategies for athletic recovery and supporting metabolic health.

## 2. Materials and Methods

### 2.1. Participants

A total of 8 healthy male university students who were regularly physically active were recruited. Regular physical activity was confirmed using the International Physical Activity Questionnaire (IPAQ) short form, with participants required to meet moderate-to-vigorous physical activity criteria (≥150 min of moderate-intensity or ≥75 min of vigorous-intensity activity per week). Inclusion criteria were: healthy males aged 20–25 years, body mass index (BMI) between 18 and 22 kg/m^2^; and having maintained at least 4 weeks of stable training prior to the study, with ≥3 sessions per week of moderate-intensity exercise (e.g., running or cycling) for at least 30 min each; and willing to adhere to the study protocol. Exclusion criteria included: history of serious illness or surgery within the past 3 months; presence of metabolic disorders such as diabetes or hyperlipidemia; use of medications or nutritional supplements (including BCAAs) within the past 6 months; intense exercise or alcohol consumption within 48 h before testing; musculoskeletal injury during the study; and participation in other strenuous exercise outside the experiment.

A priori sample size calculation was performed using G*Power software (version 3.1.9.7) based on data from a previous study investigating BCAA supplementation effects on exercise performance. With an effect size f = 0.3, α = 0.05, power (1-β) = 0.80, and correlation among repeated measures = 0.5, the calculated sample size was 16. Then we recruited 8 participants to account for potential dropouts, providing adequate statistical power for the primary outcomes. Anthropometric characteristics of the participants are presented as mean ± standard deviation (SD): age 22.5 ± 1.2 years, height 175.3 ± 2.8 cm, weight 72.4 ± 4.1 kg, BMI 21.3 ± 1.4 kg/m^2^, the percentage of body fat 16.66 ± 5.41%. All participants provided written informed consent, and the study was approved by the Ethics Committee of Beijing Sport University (approval number: 2023233H).

### 2.2. Study Design

This study employed a randomized, placebo-controlled, crossover design and was conducted between March and July 2023, with a total duration of 10 weeks including participant recruitment, experimental trials, washout periods, and preliminary analysis. The study protocol followed the CONSORT (Consolidated Standards of Reporting Trials) guidelines for randomized crossover trials. There were no changes to the outcomes or methodology after the trial commenced.

### 2.3. Randomization and Supplementation Protocol

Participants were randomly assigned using a computer-generated random number sequence to receive either BCAA or placebo in the first trial, with the order crossed over after a 7-day washout period. The BCAA supplement consisted of a leucine: isoleucine: valine mixture (2:1:1 ratio; Beijing Combest Co., Beijing, China) at a dose of 0.2 g/kg body weight per serving, twice daily (morning and evening) for three consecutive days [5,15]. This dosing strategy was consistent with previous trials demonstrating the effectiveness and safety of short-term BCAA supplementation in exercise settings [7,11]. The placebo consisted of an isocaloric corn starch powder (Beijing Combest Co., China) matched for color, texture, odor, and taste. Both supplements were dissolved in 300 mL of water in identical unlabeled containers and immediately consumed under supervision.

### 2.4. Dietary Control

Participants were instructed to record their habitual dietary patterns and intake for one week preceding the study using the Mint Health mobile application (version 2.5.1), a validated dietary assessment tool with a comprehensive food database, to assess baseline nutrition and minimize confounding factors. During the trail, their diets were strictly controlled and detailed records were maintained: participants maintained their usual diet without special dietary interventions, and the intake of any additional nutritional supplements (especially protein supplements) was prohibited. Alcohol and caffeinated beverages were avoided throughout the supplementation period to minimize confounding. all meals (including snacks) were photographed and logged daily through the Mint Health application. Total daily energy intake and macronutrient composition were analyzed to ensure consistency between trails. To minimize the influence of acute dietary intake on metabolic outcomes, all participants fasted for at least 8 h before each test day, with only water permitted. Based on these records, the average daily energy and macronutrient intakes for the BCAA group were 2082 ± 330 kcal (carbohydrates 243 ± 45 g, fat 62 ± 19 g, protein 113 ± 12 g), and for the placebo group were 2090 ± 360 kcal (carbohydrates 249 ± 43 g, fat 65 ± 18 g, protein 115 ± 14 g). No significant differences were observed in pre-exercise dietary intake between conditions, supporting dietary standardization.

### 2.5. Experimental Procedure

Two weeks prior to the experimental trials, each participant accepted dual-energy X-ray absorptiometry (iDXA, GE Healthcare/Lunar, Madison, WI, USA) to measure body composition, completed an incremental cycling test to determine their maximal oxygen uptake (VO_2_max). Following a 5 min rest when arrived at the laboratory, participants performed an incremental exercise test to volitional exhaustion on a Monark cycle ergometer (Ergomedic 839E, Monark Exercise AB, Vansbro, Sweden), beginning with a 5 min warm-up at 80 W. After a 3 min rest, the workload started at 80 W and increased by 40 W every 3 min until 200 W, thereafter increasing by 20W every 2 min until volitional exhaustion, with participants maintaining a cadence of ≥60 rpm. Expired gases were analyzed breath-by-breath using a MetaLyzer 3B system (CORTEX Biophysik GmbH, Leipzig, Germany).

On the day before each trial, participants were instructed to maintain their normal daily routines, while avoiding strenuous exercise and caffeine, and to observe an overnight fast of at least 8 h. On the test day, participants arrived at the laboratory in a fasted state, consumed the assigned supplement, and underwent baseline fingertip and venous blood samples sampling. After a 30 min rest period, additional blood samples were collected, followed by the exercise test.

The exercise protocol consisted of two consecutive stages. Stage 1 involved 60 min of constant-load cycling at 60% of VO_2_max. Stage 2 was performed immediately afterward and consisted of cycling at 80% VO_2_max until exhaustion (time to exhaustion, TTE). Throughout the test, respiratory gases were continuously monitored using a metabolic analyzer (K5, COSMED, Rome, Italy) to measure VO_2_ and VCO_2_ to assess participants’ endurance capacity. Substrate oxidation rates were calculated using the Frayn equations: carbohydrate oxidation (g/min) = 4.210 × VCO_2_ − 2.962 × VO_2_; fat oxidation (g/min) = 1.695 × VO − 1.701 × VCO_2_, to quantify metabolic efficiency during exercise. In addition, the rating of perceived exertion (RPE) and visual analog scale (VAS) scores were recorded every 15 min during Stage 1. Heart rate were continuously monitored throughout both stages. This submaximal steady-state exercise was designed to evaluate metabolic responses under moderate-intensity conditions.

Venous blood samples were collected at three time points: baseline (fasted state), 30 min after supplement ingestion (pre-exercise), and immediately after Stage 2 (post-exercise). Plasma samples were processed immediately to separate and stored at −80 °C until analysis.

**Figure 1:**
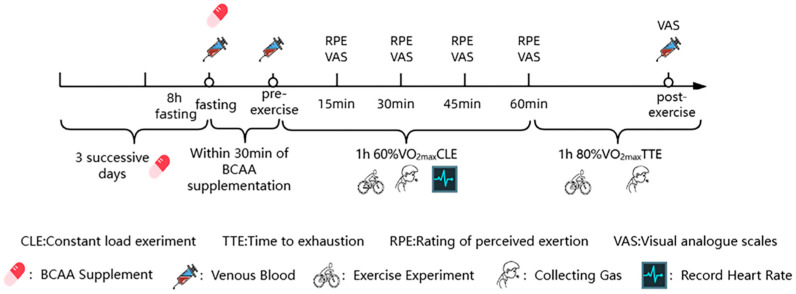
Experimental flowchart of the study protocol. Schematic overview of the supplementation, exercise. Participants underwent 8 h fasting followed by BCAA or placebo supplementation within 30 min before exercise testing. The protocol included a constant load exercise (CLE) at 60% VO_2_max for 1 h and a time-to-exhaustion (TTE) test at 80% VO_2_max. Blood sampling, gas collection, rating of perceived exertion (RPE), and visual analogue scale (VAS) assessments were performed at defined time points.

The overall experimental protocol, including the supplementation schedule, exercise test, is illustrated in Figure 1.

### 2.6. Blood Biochemical Analysis

Blood Samples were drawn into anticoagulant tubes containing sodium heparin and immediately centrifuged at 3000× *g* for 10 min at 4 °C to separate plasma and serum. Blood glucose and lactate were measured using Electrochemical analyzers (EKF Biosen C-Line, Germany). Insulin (INS), non-esterified fatty acids (NEFA), and β-hydroxybutyrate (β-HB) were quantified using enzyme-linked immunosorbent assays (Sinouk Bio, Beijing, China). Plasma ammonia was measured using an enzymatic cycling assay kit (Sinouk Bio, Beijing, China) according to the manufacturer’s instructions.

### 2.7. Metabolite Profiling and Quantification

One of eight participants withdrew due to injury before the second trail, and a plasma sample of post-exercise of one participant in placebo group due to failure in quality control criteria (e.g., abnormal signal intensity and poor feature alignment), a total of 4 samples was excluded from metabolomic analysis. Therefore, metabolomics analyses were conducted on 44 samples.

For metabolite extraction, plasma aliquots (100 µL) were mixed with 300 µL of ice-cold methanol containing a comprehensive set of internal standards (covering amino acids, organic acids, and other central carbon metabolites). After vortexing and incubation at −20 °C for 30 min to precipitate protein, the mixture was centrifuged (15,000× *g*, 15 min, 4 °C). The supernatant was transferred, dried under a gentle stream of nitrogen, and reconstituted in 100 µL of 50% methanol for LC-MS analysis.

Metabolite separation and detection were performed using ultra-high-performance liquid chromatography coupled with quadrupole time-of-flight mass spectrometry (UHPLC–QTOF–MS) in both positive and negative electrospray ionization modes. Chromatographic separation used a C18 column (2.1 × 100 mm, 1.7 µm) with water (0.1% formic acid) and acetonitrile (0.1% formic acid) as mobile phases under a 0.3 mL/min flow rate. The gradient increased from 2% to 98% B within 20 min at 40 °C. Mass spectra were acquired in the range of *m*/*z* 50–1200 with a resolution of 30,000.

Raw data were processed using Compound Discoverer 3.3 (Thermo Fisher Scientific, Waltham, MA, USA). Pre-processing included features filtering based on retention time and mass-to-charge ratio (*m*/*z*) parameters, peak alignment across samples and peak extraction using predefined mass tolerance and adduct settings. Peak areas were used for relative quantification. Metabolite identification was achieved by matching using high-resolution MS^2^ spectral libraries (mzCloud and mzVault) and the MassList MS^1^ library. Specifically, precursor ion *m*/*z* values from MS^1^ spectra were used to determine the molecular weight of metabolites. Molecular formulas were predicted based on mass accuracy (ppm error < 5) and adduct information, and subsequently matched to reference compounds in the databases. For metabolites with available MS^2^ spectra, fragment patterns and collision energy patterns were compared against reference spectra to confirm identity. To ensure data quality, only metabolites with a coefficient of variation (CV) < 30% in pooled QC samples were retained for downstream statistical analysis. Metabolite annotation confidence was assigned following the Metabolomics Standards Initiative (MSI) guidelines. Given that metabolite identification was primarily based on MS^1^ accurate mass matching, all reported annotations are considered tentative unless supported by MS/MS spectral matching (Level 2 or higher according to Metabolomics Standards Initiative guidelines). Lipid species are reported as sum compositions (e.g., PC 36:3) rather than specific acyl-chain assignments unless confirmed by orthogonal methods.

### 2.8. Quality Control and Differential Metabolites Screening

Pooled quality control (QC) samples were prepared by combining equal aliquots from all samples and were analyzed periodically to monitor system stability. Before sample analysis, three QC injections were run before the analytical sequence to equilibrate the system, and one QC sample was subsequently injected after every six samples throughout the acquisition run. The injection sequence was randomized. To assess analytical reproducibility, the processed data (after normalization and filtering) was unit-variance-scaled (UV-scaled) and subjected to unsupervised Principal Component Analysis (PCA). The tight clustering of the QC samples in the PCA score plot, confirmed the high stability and reproducibility of the analytical platform throughout the sequence.

Orthogonal partial Least Squares-Discriminant Analysis (OPLS-DA) was employed to discriminate metabolic profiles between BCAA and placebo groups. The models were constructed using seven-fold cross-validation (or k = 2n for groups with ≤3 biological replicates per condition) to obtain the model parameters R^2^ and Q^2^. Higher R^2^ and Q^2^ values (closer to 1.0) indicate greater model stability and predictive reliability. To further assess model robustness and exclude overfitting, 200 random permutation tests were performed, in which class labels were randomly reassigned, and new models were built for each permutation. Regression lines of permuted R^2^ and Q^2^ values against the correlation coefficient were then generated. Models were considered valid and not overfitted when R^2^ > Q^2^ and the Q^2^-intercept was below zero. This approach ensured that the resulting OPLS-DA models accurately reflected biological differences rather than random variation. Differential metabolites were identified based on the following criteria: variable importance in projection (VIP) ≥ 1.0 from the OPLS-DA model, fold change (FC) > 1.2 or < 0.83, and adjusted *p*-value < 0.05 (paired *t*-test with Benjamini–Hochberg false discovery rate correction). Metabolites satisfying all three criteria were considered statistically significant.

### 2.9. Statistical Analyses

Physiological and biochemical outcomes are expressed as mean ± standard deviation (SD). Normality was assessed using the Shapiro–Wilk test; non-normally distributed variables were log_10_-transformed prior to analysis. A two-way repeated-measures ANOVA (condition × time) was performed, with Greenhouse-Geisser correction when sphericity was violated. Post hoc comparisons were adjusted using the Bonferroni method. For datasets with missing values or unbalanced observations, a linear mixed-effects model with subject as random intercept was employed.

Metabolomics data were normalized by total ion current before analysis. Group differences were evaluated using the OPLS-DA and paired *t*-test approach described in Section 2.8. Spearman’s rank correlation was used to examine associations between significant metabolites and physiological outcomes, with false discovery rate (FDR) correction applied for multiple testing. All analyses were conducted in R (version 4.3.0) and MetaboAnalyst 6.0.

## 3. Results

### 3.1. Metabolomics Results

#### 3.1.1. Data Quality Control

QC samples were employed to evaluate the reproducibility and stability of the metabolomics data. The Pearson correlation coefficients for QC samples, based on relative quantification of metabolites, exceeded 0.9 in both positive- and negative-ion modes, confirming high analytical reproducibility. The PCA scores plots showed trends of group separation, visualizing the inherent metabolic differences between the BCAA and placebo groups across pre- and post-exercise time points (Supplementary Figure S1).

The OPLS-DA models constructed for each comparison demonstrated clear discrimination between BCAA and placebo groups. All model parameters exhibited satisfactory performance (R^2^Y > 0.7, Q^2^ > 0.4), and permutation tests (200 iterations) confirmed that all models were statistically valid without overfitting (Q^2^-intercept < 0).

#### 3.1.2. Characteristics of Differential Metabolite Between Supplementation Groups

Identifications are tentative and based on MS^1^ accurate mass; lipid annotations are presented as sum compositions to reflect the confidence level. Following quality filtering, a total of 641 and 473 metabolites were identified in positive- and negative-ion modes, respectively. Differential analysis revealed significant changes following BCAA supplementation. In the pre-exercise comparison (B_BCAA_g vs. B_Placebo_g), 40 metabolites in positive-ion mode (21 up- and 19 down-regulated) and 45 in negative-ion mode (35 up- and 10 down-regulated) were significantly altered (Top 20 metabolites in Supplementary Table S1). Volcano plots and heatmaps highlighted key alterations between BCAA and placebo groups (Supplementary Figures S2A,B and S3A,B). Overall, BCAA intake induced pronounced alterations in metabolite profiles compared to placebo, particularly elevating metabolites involved in lipid and amino acid metabolic pathways.

#### 3.1.3. Identification of Core Metabolites Altered by Exercise and BCAAs Supplementation

We focused on the core metabolites altered by BCAA supplementation before and after exercise (Supplementary Figures S2C,D and S3C,D, Top 20 metabolites in Supplementary Table S2). Multiple ranking methods (by *p*-value, fold change, and VIP score) consistently identified carnitine, fatty acid esters of hydroxy fatty acids (FAHFA), and tryptophan-related metabolites as highly influential variables (Supplementary Figure S4).

**Figure 2:**
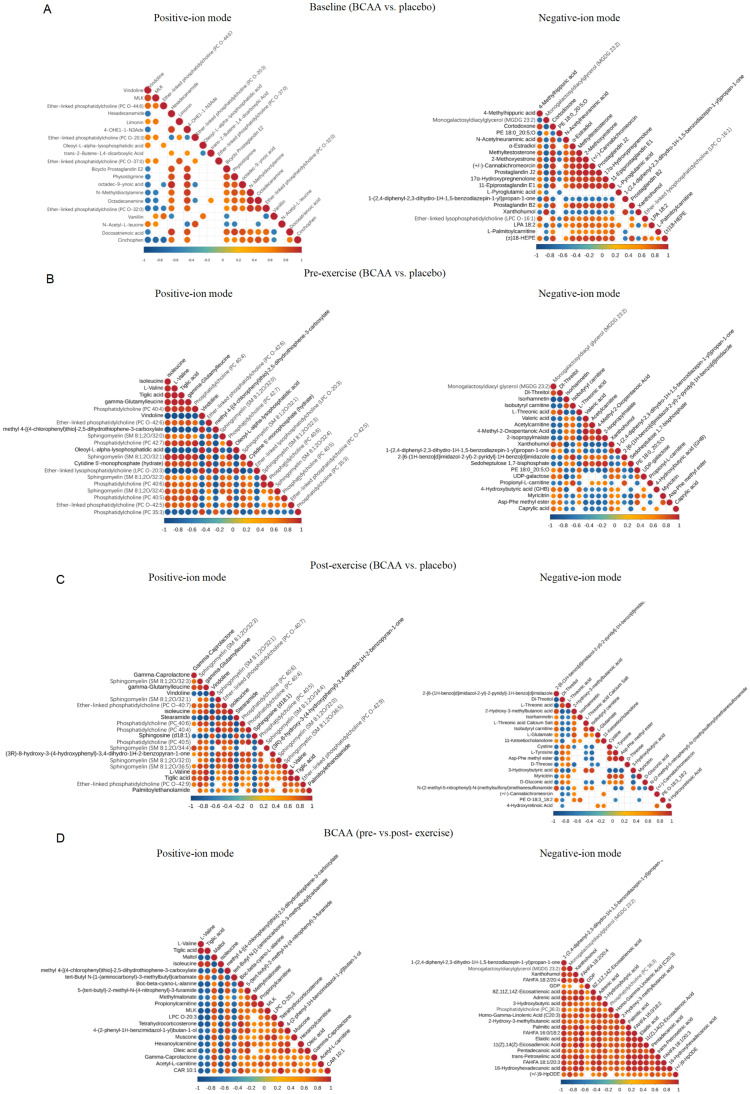
Correlation analysis of core differential metabolites under positive- and negative-ion modes. Correlation heatmaps show Pearson correlation coefficients among core differential metabolites (VIP > 1, *p* < 0.05) identified under different experimental conditions. Results are shown for positive-ion mode (left panels) and negative-ion mode (right panels). (**A**) Baseline comparison between BCAA and placebo groups. (**B**) Pre-exercise comparison between BCAA and placebo groups. (**C**) Post-exercise comparison between BCAA and placebo groups. (**D**) Comparison of post- versus pre-exercise under BCAA supplementation. Color intensity represents the strength and direction of correlations, with red indicating positive correlations and blue indicating negative correlations. Only statistically significant correlations (*p* < 0.05) are displayed. Abbreviations: BCAA, branched-chain amino acid; VIP, variable importance in projection.

Pearson correlation analysis was performed to examine inter-relationships among these core metabolites (Figure 2A–D). Heatmaps depicting correlations among the top 20 positive- and negative-ion metabolites in the BCAA vs. placebo at pre-exercise (Figure 2B) and post-exercise (Figure 2C) further illustrated coordinated metabolic changes. For instance, in the pre-exercise comparison, negative-ion metabolites such as acetylcarnitine, xanthohumol, and myricitrin strongly positively correlated, suggesting that BCAA supplementation can coordinate the expression of multiple functional metabolites prior to exercise. In the post-exercise comparison, correlations were observed among lipids (e.g., stearamide, various PCs) and amino-acid metabolites (e.g., γ-glutamylleucine) in the positive-ion mode. In the pre- vs. post-exercise comparison under BCAA supplementation, the top 20 negative-ion metabolites exhibited strong positive correlation coefficients, indicating potential synergistic interactions—a pattern not observed for positive-ion metabolites (Figure 2D).

#### 3.1.4. Pathway Enrichment Analysis

**Figure 3:**
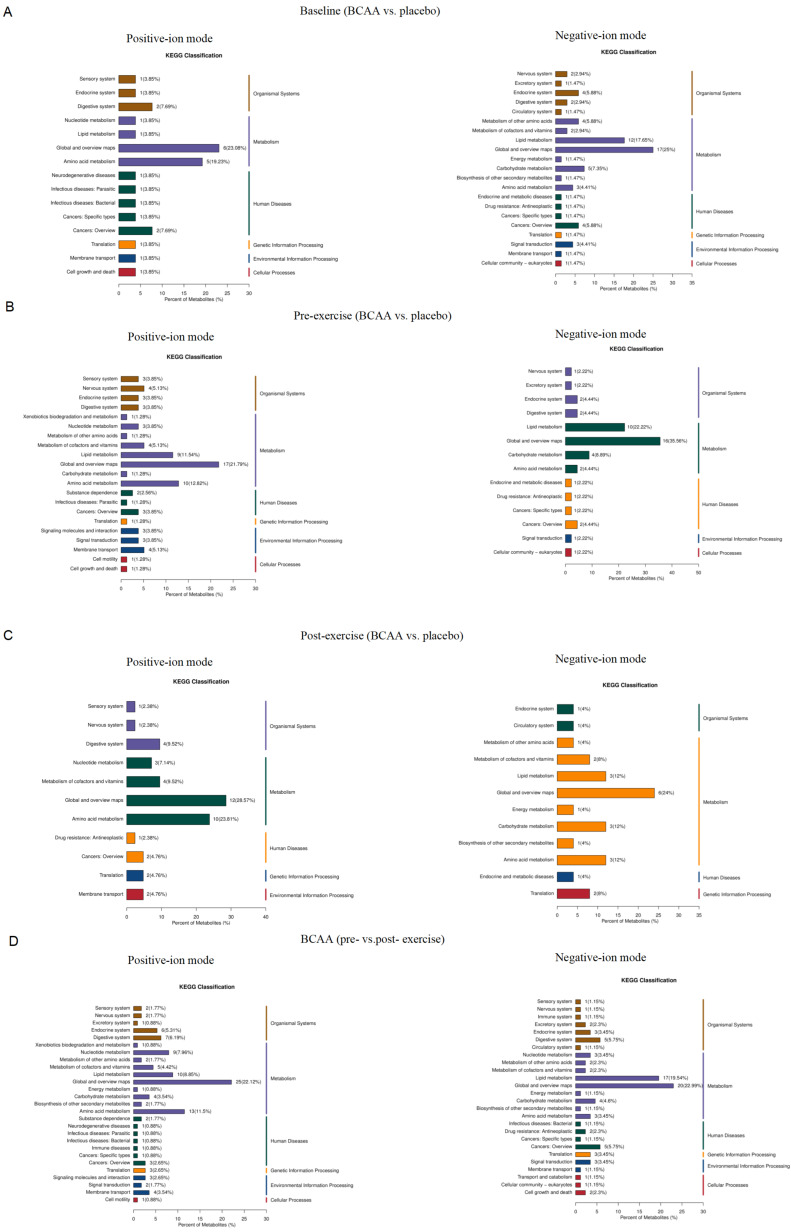
KEGG pathway classification of differential metabolites. Bar charts show the number (and percentage) of metabolites in each major KEGG category. (**A**) Baseline comparison (BCAA vs. Placebo). (**B**) Post-exercise comparison between BCAA and placebo groups. (**C**) Pre-exercise comparison between BCAA and placebo groups. (**D**) Comparison of post- versus pre-exercise under BCAA supplementation. For each comparison, the left panel represents positive-ion mode and the right panel represents negative-ion mode. Values in bars indicate counts (percent in parentheses). Abbreviations: KEGG, Kyoto Encyclopedia of Genes and Genomes. BCAA, branched-chain amino acid.

To further explore the biological functions of the differential metabolites, KEGG pathway annotation and classification were performed on the differential metabolites identified between post-exercise BCAA and Placebo groups. As shown in Figure 3A, most metabolites were annotated to “Metabolism” pathways, with notable enrichment in sub-pathways, such as “Amino Acid Metabolism,” “Lipid Metabolism,” and “Vitamin Metabolism.” Several metabolites were also associated with biological processes related to “Signal Transduction,” “Immune System,” and “Endocrine System”.

In the pre-exercise comparison, both positive- and negative-ion metabolites were enriched in “Lipid Metabolism” and “Amino Acid Metabolism” pathways (Figure 3B). In the post-exercise comparison, positive-ion metabolites were most enriched in “Amino Acid Metabolism,” whereas negative-ion metabolites were most enriched in “Lipid Metabolism,” “Carbohydrate Metabolism,” and “Amino Acid Metabolism” (Figure 3C). In addition, KEGG classification of metabolites altered before and after exercise within the BCAA group is shown in Figure 3D.

### 3.2. Integrative Correlation Analysis

#### 3.2.1. Correlation Between Blood Biochemical Markers and Metabolites

**Figure 4:**
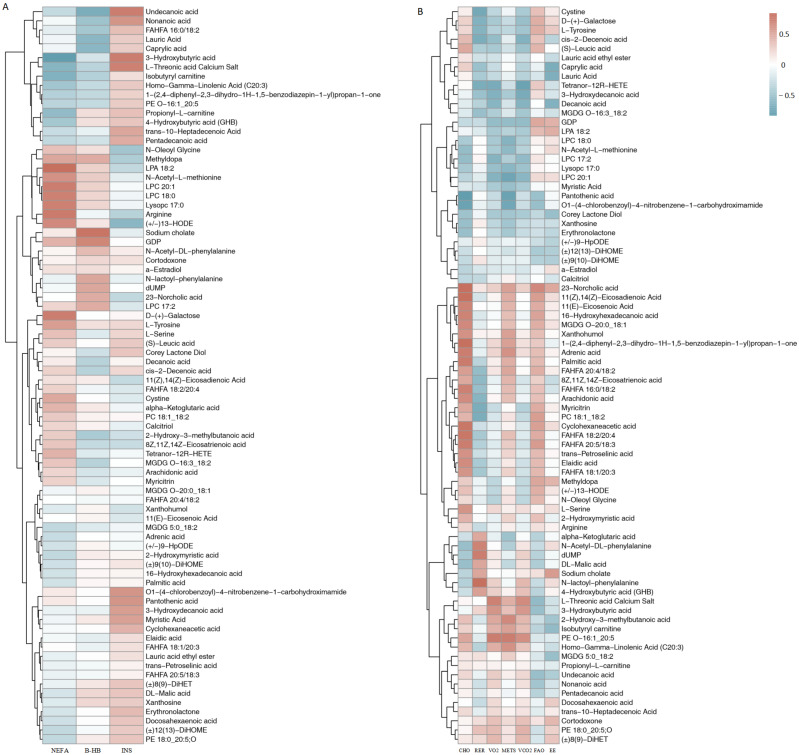
Correlation of physiological parameters with differential metabolites under BCAA supplementation. Heatmaps show Pearson correlation coefficients (r) between (**A**) blood biochemical markers (NEFA, β-HB, INS) and metabolite levels, and (**B**) exercise parameters (FAO, VO_2_, RER, EE, CHO, METS, VCO_2_) vs. metabolites. Color indicates value (red = positive, blue = negative). Abbreviations: NEFA, non-esterified fatty acid; β-HB, β-hydroxybutyrate; INS, insulin; CHO, carbohydrate oxidation; RER, respiratory exchange ratio; VO_2_, oxygen uptake; METS, metabolic equivalents; VCO_2_, carbon dioxide output; FAO, fatty acid oxidation; EE, energy expenditure.

Spearman correlation analysis (Figure 4A) revealed that NEFA levels were significantly positively correlated with anti-inflammatory FAHFA metabolites (FAHFA 16:0/18:2 and FAHFA 20:4/18:2; r = 0.65, *p* < 0.01). β-HB was positively correlated with caprylic acid (r = 0.61, C<0.05) and lauric acid (r = 0.58, *p* < 0.05). Insulin was negatively correlated with phosphatidylcholine (PC) 18:1_18:2 (r = −0.62) and lysophosphatidylcholine (LPC) 20:1 (r = −0.59).

#### 3.2.2. Correlation Between Respiratory Gas and Metabolites

FAO rate showed a strong positive correlation with propionyl-L-carnitine (r = 0.72, *p* < 0.01) and monogalactosyldiacylglycerol (MGDG) 5:0_18:2 (r = 0.68, *p* < 0.01). VO_2_ was positively correlated with sodium cholate (r = 0.64, *p* < 0.05) and xanthohumol (r = 0.67, *p* < 0.01). The RER was negatively correlated with FAHFA and polyunsaturated fatty acid (PUFA) lipid metabolites (r ≈ −0.58 to −0.65, *p* < 0.05), indicating a shift in substrate utilization toward fat oxidation (Figure 4B).

### 3.3. Physiological and Biochemical Outcomes

During steady-state cycling, BCAA supplementation modified substrate utilization and metabolic byproducts compared to placebo (Supplementary Figure S5). The RER was significantly lower in the BCAA group at the end of exercise (0.85 ± 0.05 at 60 min vs. 0.92 ± 0.04 for placebo, *p* < 0.05), indicating greater reliance on fat oxidation. Consistent with enhanced lipid catabolism, circulating β-HB concentrations were higher in the BCAA condition (0.07 ± 0.03 mmol/L) than with placebo (0.05 ± 0.03 mmol/L). NEFA levels also tended to be elevated post-exercise in the BCAA group (0.62 ± 0.25 mmol/L vs. 0.59 ± 0.26 mmol/L), reflecting increased lipolysis, although this difference was not statistically significant. No significant differences were observed in plasma glucose or lactate concentrations between the BCAA and placebo conditions, either pre- or post-exercise.

BCAA intake additionally influenced hormonal and fatigue-related biochemical markers. Thirty minutes after ingestion (at pre-exercise time point), plasma insulin increased modestly in the BCAA group (from ~13–14 μIU/mL to ~15 μIU/mL), consistent with the insulinotropic effect of leucine, whereas no substantial change occurred with placebo. By the end of exercise (60 min), insulin declined to comparable levels across conditions (15 ± 1 μIU/mL), indicating that the transient nature of the BCAA-induced increase. Blood ammonia, a fatigue-associated metabolite, increased in both groups but was slightly attenuated with BCAA supplementation, peaking at 23.8 ± 1.5 μmol/L versus 24.5 ± 1.7 μmol/L with placebo.

## 4. Discussion

In this study, BCAA supplementation during endurance exercise significantly reduced RER, increased circulating β-HB, and tended to elevate NEFA compared with placebo, collectively indicating enhanced the effect of fat oxidation. Ammonia accumulation was modestly attenuated, while insulin dynamics remained stable, reflecting a shift in energy substrate utilization. These physiological and biochemical outcomes provide macroscopic evidence that aligns with and complements the metabolomic findings.

### 4.1. BCAA Supplementation Remodels Lipid and Energy Metabolism

The results in this study showed that the supplement of BCAAs influenced metabolism both at rest and after exercise. In the BCAA group at post-exercise time point, the marked upregulation of pantetheine (log_2_FC = +2.3) and DL-carnitine suggests potential mechanistic insights: first, pantetheine is a precursor of Coenzyme A, and its increase may reflect the elevated TCA cycle flux, while the elevating of DL-carnitine indicates the enhancing of fatty acid β-oxidation, consistent with the increased energy demand after exercise [5]. Second, the significant alterations in phosphatidylethanolamine (PC 36:3) and stearamide (VIP > 2.5) further suggest that BCAAs may influence phospholipid metabolism [16]. Compared to the placebo group, the 61 metabolites upregulated in the BCAA group indicate that BCAA combined with exercise elicits a broader metabolic response, including the modulation of lipid metabolism pathways (e.g., PC 40:5) and the changes in antioxidant-related metabolites (e.g., xanthohumol). In the pre-exercise comparison, BCAA intake upregulated 46 metabolites, with pronounced downregulation of tryptophan metabolites (e.g., kynurenic acid). These results support the “BCAA–tryptophan competition” hypothesis, potentially providing a molecular basis for the reported effects of BCAAs on central fatigue, though future studies incorporating cerebrospinal fluid metabolomics would be valuable to confirm this mechanism.

### 4.2. Dual-Ion Mode Metabolic Regulation by BCAAs

Our study showed that BCAAs affected metabolic profiles in both positive- and negative-ion modes. Integration analysis revealed that negative-ion metabolites exhibited synergistic patterns: the top 20 negative-ion metabolites in the BCAA group at post-exercise time point were highly positively correlated, exemplified by the synchronous increase in monogalactosyldiacylglycerol (MGDG 23:2) and sodium cholate, which may jointly influence the metabolism of bile acid and the absorption of lipid. Hypoxanthine, the most significantly downregulated metabolite and an end product of purine metabolism, suggests BCAAs may modulate exercise-induced metabolic stress by affecting ATP degradation pathways. KEGG analysis showed that, in the positive-ion mode, amino acid metabolism predominated, especially enrichment of BCAA degradation pathways (e.g., valine/leucine degradation), confirming their role as a “metabolic switch”; in the negative-ion mode, significant enrichment of carbohydrate metabolism pathways may reflect the maintenance of blood glucose homeostasis by BCAAs via gluconeogenic precursors (such as alanine) [13], indicating the mode-dependent metabolic effects of BCAAs [17], providing further evidence for the role of BCAAs as energy substrates during endurance exercise.

### 4.3. Systemic Metabolic Coordination: Evidence from Correlation Analysis

This study integrates blood biochemical indicators, respiratory metabolic indicators, and metabolomic data to construct a “metabolic interaction network” mediated by BCAA supplementation. The correlations indicate that BCAAs contributed to beneficial exercise- induced changes within this networks. Specifically, among blood biochemical markers, NEFA was strong positive correlation (r > 0.6) with various anti-inflammatory FAHFA lipids (such as FAHFA 16:0/18:2 and FAHFA 20:4/18:2), suggesting that the mobilization of fat may be accompanied by the synthesis of anti-inflammatory lipids mediators—a finding consistent with studies noting the dual role of FAHFAs in lipid oxidation and inflammation regulation [10]. β-HB was positively correlated with medium-chain fatty acids (caprylic acid and lauric acid) [5], reflecting the co-activating effect of fat oxidation and ketogenesis under BCAA supplementation, which aligns with an enhanced mitochondrial β-oxidation model. Meanwhile, insulin was negatively correlated (r = −0.58 to −0.65) with several phospholipid metabolites (including PC 36:3, lysophosphatidylcholine (LPC 20:1), and LPC 17:2), suggesting that BCAA supplementation may influence insulin signaling through the modulation of membrane lipid metabolism, a notion supported by established links between dysregulated lipid metabolism and insulin resistance [9]. In addition, the changes in choline metabolites further implied a tight coupling between membrane stability and energy metabolism.

Regarding respiratory gas parameters, FAO was strongly positively correlated (r > 0.7) with carnitine-related metabolites (propionyl-L-carnitine) and MGDG-class lipids (MGDG 23:2) [18], indicating that enhanced FAO coincides with improved carnitine-mediated mitochondrial lipid transport. VO_2_ was positively correlated with sodium cholate and the antioxidant polyphenol xanthohumol, suggesting that under BCAA supplementation, the increasing of aerobic capacity is accompanied by the enhancing of oxidative stress defense, consistent with models of AMPK activation and increased metabolic flexibility [19]. The negative correlation between RER and FAHFA/PUFA metabolites, further confirms that BCAA promotes substrate utilization efficiency during exercise by enhancing lipid oxidation [12]. These correlations are in agreement with the results of physiological observations, where the lowering of RER and the elevating of β-HB in the BCAA group reflected the enhancing of fat oxidation, and the attenuated rise in blood ammonia was consistent with the reduction in purine degradation products such as hypoxanthine.

Overall, blood indicators, respiratory data, and metabolomics were highly coupled, forming an integrated metabolic interaction network [20]. BCAAs appear to regulate post-exercise energy metabolism in multiple ways by promoting the fat mobilization, enhancing FAO, improving the carnitine system, modulating membrane phospholipid metabolism. This systemic metabolic adaptation supports the rationale for using BCAAs as exercise nutritional supplements [21].

### 4.4. Strengths and Limitations

A key strength of this study is the integration of conventional physiological and biochemical indicators (e.g., RER, β-HB, NEFA, ammonia) with comprehensive metabolomic profiling, providing a multi-level perspective on BCAAs-mediated modulation of exercise metabolism. Pearson correlation analysis and PCA of QC samples confirmed data repeatability and clear group trends, ensuring the reliability of subsequent differential metabolite analysis [22]. Correlating metabolite changes with key physiological, biochemical, and respiratory gas parameters helped integrate molecular shifts with physiological functions [23].

However, this study has several limitations that should be considered. First, the sample size was limited (n = 44 samples from 8 participants), which may reduce the statistical power of subgroup analyses. Second, participants were healthy young males, limiting the generalizability of findings to other populations, such as females, older adults, or clinical populations [24]. Third, all reported associations in this study remain correlative in nature; therefore, no causal or mechanistic conclusions can be drawn without additional targeted validation. Future studies designed to address these limitations will be essential to validate and extend these preliminary findings.

## 5. Conclusions

This study demonstrates that short-term supplementation with BCAAs significantly modulates post-exercise metabolism through two primary mechanisms: enhancing FAO to meet energy demands and improving cell membrane stability to mitigate exercise-induced stress. These effects appear to be achieved via coordinated regulation of multiple metabolic pathways, including glucose, lipid, and amino acid metabolism. The findings suggest that BCAAs act not only as energy substrates but may also contribute to broader metabolic adaptation to exercise. Overall, the findings indicate that BCAAs are associated with multifaceted metabolic effects as sports nutrition supplements and provide valuable scientific evidence supporting their potential for individualized application in precision exercise nutrition strategies. It should be noted that conclusions regarding glycogen replenishment and muscle repair are not directly supported by our data, as muscle glycogen and specific muscle damage biomarkers were not measured. Future studies incorporating these measurements would provide more definitive evidence.

## Data Availability

The raw metabolomics data (LC–MS files) generated in this study have been deposited in Zenodo and are publicly available at https://doi.org/10.5281/zenodo.17909684 (accessed on 19 December 2025).. Additional processed data matrices and annotation tables are included in the repository. Any additional data generated or analyzed during the current study are available from the corresponding author upon reasonable request.

## Supplementary Materials

The following supporting information can be downloaded at https://www.mdpi.com/article/10.3390/metabo16010041/s1. The supplementary materials include additional metabolomics and physiological data supporting the results of this study. Supplementary Figure S1: Quality control assessment and PCA score plots of metabolomic data; Supplementary Figure S2: Volcano plots of differential metabolites between BCAA and placebo groups under different exercise conditions; Supplementary Figure S3: Heatmap visualization of differential metabolites under BCAA and placebo conditions; Supplementary Figure S4: Ranking of key differential metabolites under BCAA and placebo conditions; Supplementary Figure S5: Physiological and biochemical responses to BCAA supplementation during steady-state cycling; Supplementary Table S1: Top 20 of key differential metabolites before exercise under BCAA supplementation (log_2_ fold change); Supplementary Table S2: Top 20 of key differential metabolites after exercise under BCAA supplementation (log_2_ fold change).
