# Supplementary material for: The Effect of Acute Supplementation of Branched Chain Amino Acids on Serum Metabolites During Endurance Exercise in Healthy Young Males: An Integrative Metabolomics and Correlation Analysis Based on a Randomized Crossover Study
Source: Metabolites. 2026 Jan 2;16(1):41. doi: 10.3390/metabo16010041 (PMC12844224; doi:10.3390/metabo16010041)
Supplement: Supplementary file 1 [file metabolites-16-00041-s001.zip › Supplementary Figures and Tables 20251225.pdf]

Supplementary Figures and Tables

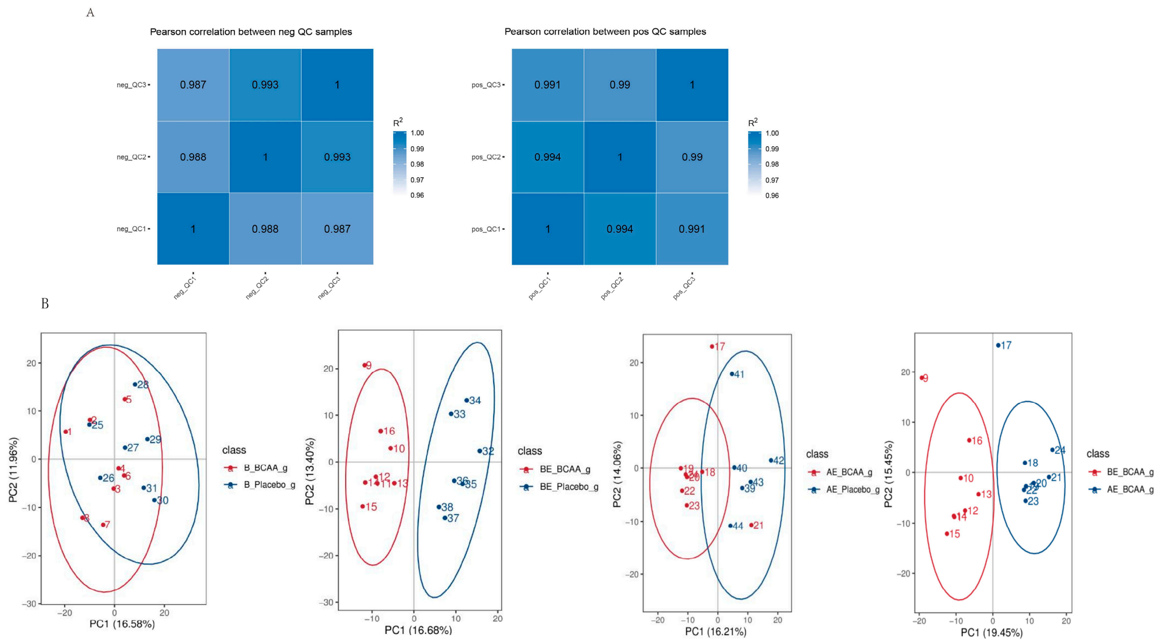

Supplementary Figure S1. Quality control assessment and PCA score plots of metabolomic data.
(A) Pearson correlation matrix for quality-control (QC) samples in negative (neg) and positive (pos) ion modes, showing all pairwise R^2 values >0.9.
(B) PCA score plots for groups in positive-ion modes, illustrating group-specific clustering trends. Axes show the percentage of variance explained.
Abbreviations: B, baseline; BE, before exercise; AE, after exercise; QC, quality control; PC, principal component; BCAA, branched-chain amino acid.

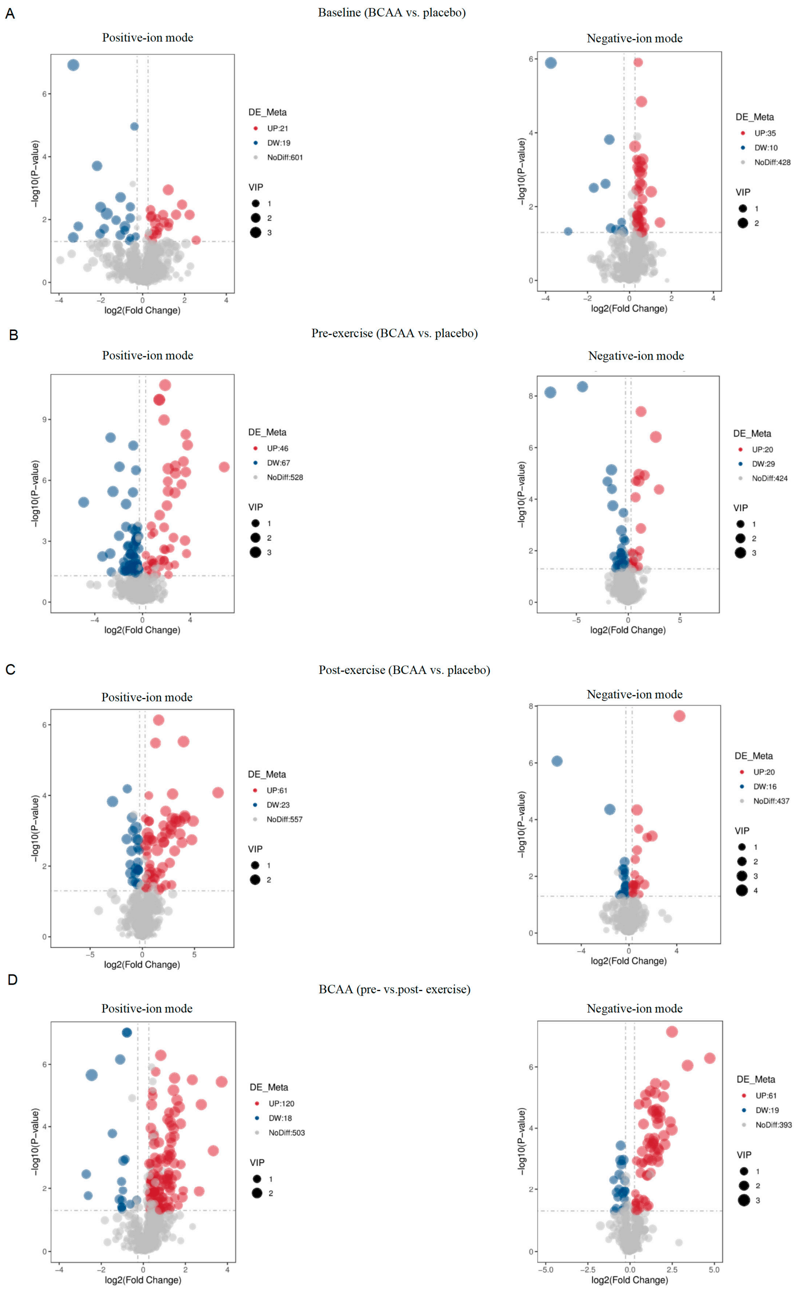

Supplementary Figure S2. Volcano plots of differential metabolites between BCAA and placebo groups under different exercise conditions.

Volcano plots show differential metabolites identified under different experimental conditions in positive- and negative-ion mode.

(A) Baseline comparison between BCAA and placebo groups.

(B) Pre-exercise comparison between BCAA and placebo groups.

(C) Post-exercise comparison between BCAA and placebo groups.

(D) Comparison of post- versus pre-exercise under BCAA supplementation.

Each dot represents an individual metabolite. The x-axis indicates $\log_2$(fold change), and the y-axis indicates $-\log_{10}$(p-value). Metabolites significantly increased are shown in red, those significantly decreased are shown in blue, and non-significant metabolites are shown in gray. Dot size reflects the variable importance in projection (VIP) score derived from OPLS-DA models.

Abbreviations: B, baseline; BE, before exercise; AE, after exercise; BCAA, branched-chain amino acid; FC, fold change; VIP, variable importance in projection.

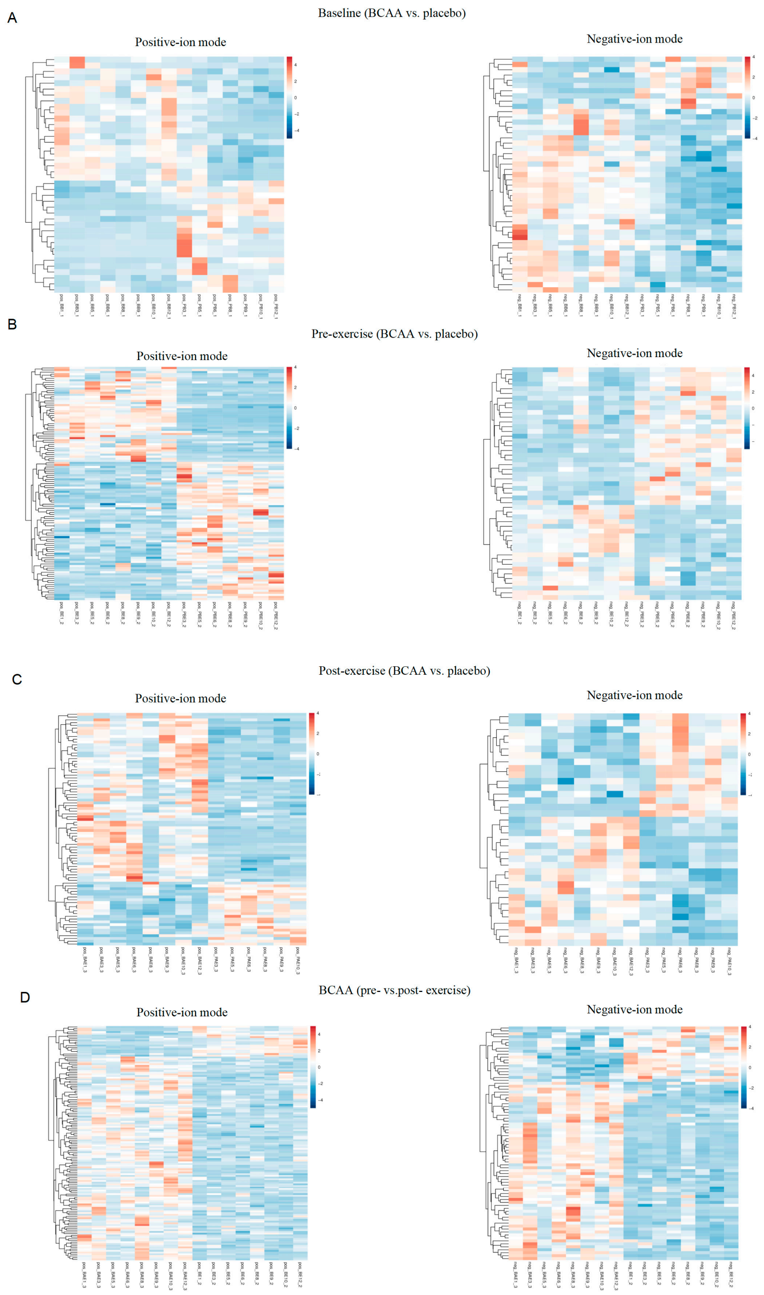

Supplementary Figure S3. Heatmaps visualization of differential metabolites under BCAA and placebo conditions.

Heatmaps depict the relative abundance patterns of differential metabolites identified under different experimental conditions in positive- and negative-ion mode.

(A) Baseline comparison between BCAA and placebo groups.

(B) Pre-exercise comparison between BCAA and placebo groups.

(C) Post-exercise comparison between BCAA and placebo groups.

(D) Comparison of post- versus pre-exercise under BCAA supplementation.

Each row represents an individual metabolite and each column represents a sample. Metabolite intensities were normalized and scaled to Z-scores prior to visualization. Hierarchical clustering was applied to metabolites to group those with similar abundance patterns. Color scale indicates relative metabolite abundance, with red representing higher and blue representing lower normalized levels.

Abbreviations: BCAA, branched-chain amino acid

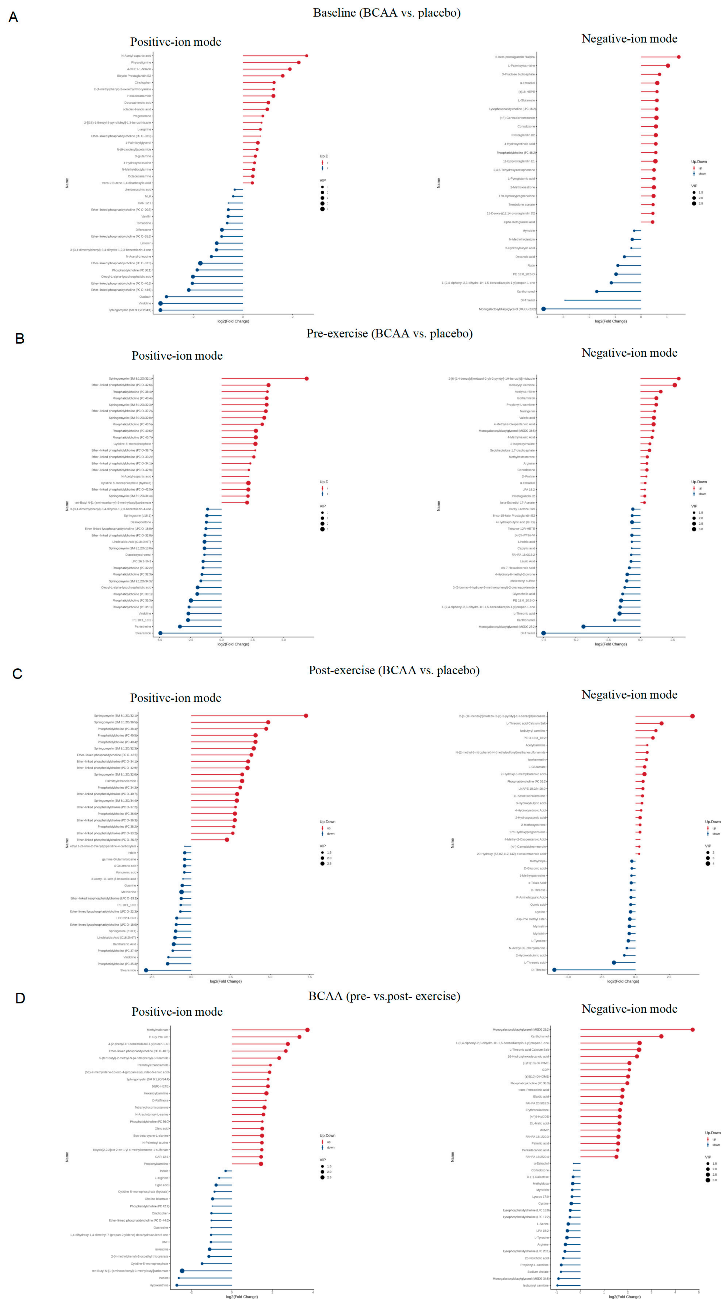

Supplementary Figure S4. Ranked of key differential metabolites under BCAA and placebo conditions.

Plots show the top-ranked differential metabolites identified under different experimental conditions in positive- and negative-ion mode, ranked by the magnitude of $\log_2$(fold change).

(A) Baseline comparison between BCAA and placebo groups.

(B) Pre-exercise comparison between BCAA and placebo groups.
(C) Post-exercise comparison between BCAA and placebo groups.
(D) Comparison of post- versus pre-exercise under BCAA supplementation.
Red bars indicate metabolites with higher abundance, whereas blue bars indicate metabolites with lower abundance in the comparison shown. Dot size reflects the variable importance in projection (VIP) score derived from OPLS-DA models.
Abbreviations: BCAA, branched-chain amino acid; FC, fold change; VIP, variable importance in projection.

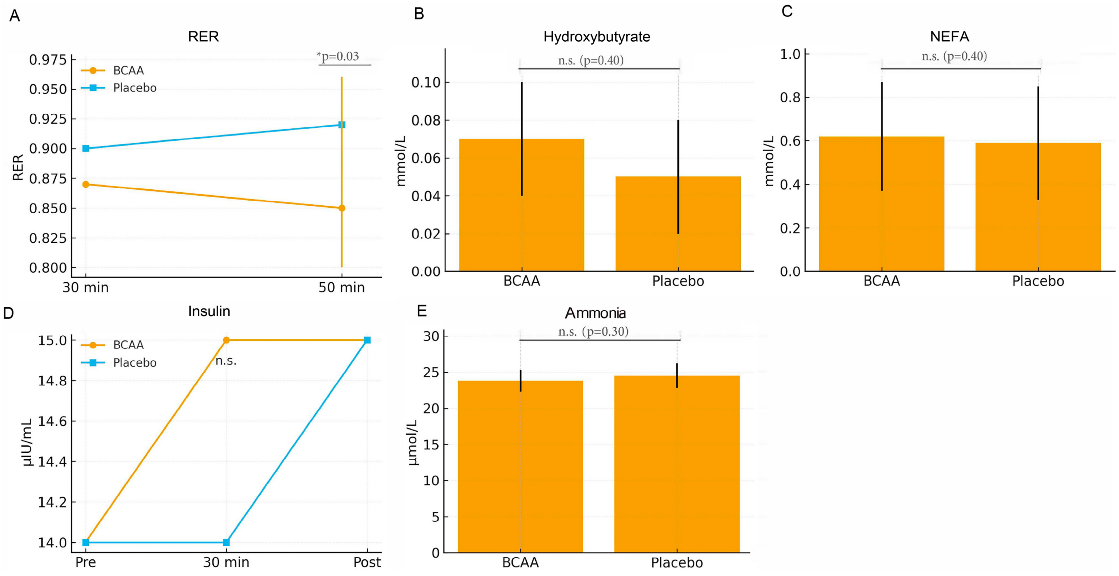

Supplementary Figure S5. Physiological and biochemical responses to BCAA supplementation during steady-state cycling.
(A) Respiratory exchange ratio (RER) measured at 30 and 50 min of steady-state exercise.
(B) Plasma β-hydroxybutyrate (β-HB) concentrations at post-exercise.
(C) Plasma non-esterified fatty acids (NEFA) concentrations at post-exercise.
(D) Plasma insulin concentrations measured at pre-exercise, 30 min, and immediately post-exercise.
(E) Plasma ammonia concentrations measured immediately post-exercise. Bars represent mean ± SD. Between-condition comparisons are annotated with p-values; only RER at 50 min reached statistical significance ($P < 0.05$), whereas other markers showed non-significant tendencies. BCAA， branched-chain amino acid.
Abbreviations: BCAA, branched-chain amino acid; RER, Respiratory exchange ratio; β-HB, β-hydroxybutyrate; NEFA, non-esterified fatty acids.

Supplementary Table S1. Top 20 of Key Differential Metabolites before Exercise under BCAA Supplementation (log_2 Fold Change)

Metabolite Name	MSI Level	MS/MS Evidence	log_2 Fold Change (BCAA vs. Placebo)	q-value (FDR)
Dl-Threitol	3	Database match (no MS/MS)	+3.77	1.13×10^{-6}
Sphingomyelin (SM 8:1;2O/32:1)	3	Database match (no MS/MS)	+3.77	1.13×10^{-6}
Stearamide	2	mzCloud MS/MS full match	−3.75	4.17×10^{-2}
Monogalactosyldiacylglycerol (MGDG 23:2)	3	Database match (no MS/MS)	+3.71	5.73×10^{-7}
Ether-linked phosphatidylcholine (PC O-42:6)	3	Database match (no MS/MS)	+3.68	1.19×10^{-5}
Phosphatidylcholine (PC 38:4)	3	Database match (no MS/MS)	+3.68	3.26×10^{-2}
Phosphatidylcholine (PC 40:4)	3	Database match (no MS/MS)	+3.62	5.32×10^{-7}
Sphingomyelin (SM 8:1;2O/32:3)	3	Database match (no MS/MS)	+3.62	1.19×10^{-5}
Ether-linked phosphatidylcholine (PC O-37:2)	3	Database match (no MS/MS)	+3.56	9.50×10^{-3}
Sphingomyelin (SM 8:1;2O/32:0)	3	Database match (no MS/MS)	+3.43	5.76×10^{-6}
Pantetheine	3	Database match (no MS/MS)	−3.36	4.17×10^{-2}
Phosphatidylcholine (PC 40:5)	3	Database match (no MS/MS)	+3.27	4.17×10^{-5}
2-[6-(1H-benzo[d]imidazol-2-yl)-2-pyridyl]-1H-…	3	Database match (no MS/MS)	+2.97	6.66×10^{-4}
Phosphatidylcholine (PC 40:6)	3	Database match (no MS/MS)	+2.76	1.31×10^{-5}
Phosphatidylcholine (PC 42:7)	3	Database match (no MS/MS)	+2.75	8.60×10^{-6}
Phosphatidylcholine (PC 36:3)	3	Database match (no MS/MS)	+2.72	9.25×10^{-5}
Lysophosphatidylcholine (LPC 17:2)	3	Database match (no MS/MS)	−2.71	3.26×10^{-2}
Ether-linked phosphatidylcholine (PC O-40:7)	3	Database match (no MS/MS)	+2.70	8.04×10^{-2}

Metabolite Name	MSI Level	MS/MS Evidence	$\log_2$ Fold Change (BCAA vs. Placebo)	q-value (FDR)
Hypoxanthine	2	mzCloud MS/MS full match	−2.68	5.73×10^{-7}
Sodium cholate	3	Database match (no MS/MS)	+2.65	1.19×10^{-5}

Key metabolites identified before exercise following BCAA supplementation, including lipid-related metabolites, carnitine-associated metabolites, and other representative significantly altered metabolites, are listed in this table. Metabolites were selected based on a fold change (FC) ≥ 1.2 or ≤ 0.83 and a Benjamini–Hochberg false discovery rate (FDR)-adjusted q-value < 0.1. Identification details, effect sizes ($\log_2$ fold change), and FDR-adjusted q-values are provided.

Supplementary Table S2. Top 20 of Key Differential Metabolites after Exercise with BCAA Supplementation ($\log_2$ Fold Change).

Metabolite Name	MSI Level	MS/MS Evidence	$\log_2$ Fold Change (BCAA vs. Placebo)	q-value (FDR)
Sphingomyelin (SM 8:1;2O/32:1)	3	Database match (no MS/MS)	+4.07	0.0169
Dl-Threitol	3	Database match (no MS/MS)	+4.06	0.0169
Sphingomyelin (SM 8:1;2O/36:5)	3	Database match (no MS/MS)	+4.06	0.0169
Phosphatidylcholine (PC 38:4)	3	Database match (no MS/MS)	+3.95	0.0007
2-[6-(1H-benzo[d]imidazol-2-yl)-2-pyridyl]-1H-…	3	Database match (no MS/MS)	+3.81	0.0324
Phosphatidylcholine (PC 40:5)	3	Database match (no MS/MS)	+3.60	0.0265
Phosphatidylcholine (PC 40:4)	3	Database match (no MS/MS)	+3.56	0.0169
Sphingomyelin (SM 8:1;2O/32:3)	3	Database match (no MS/MS)	+3.22	0.0169
Ether-linked phosphatidylcholine (PC O-42:6)	3	Database match (no MS/MS)	+3.22	0.0169
Ether-linked phosphatidylcholine (PC O-34:1)	3	Database match (no MS/MS)	+3.10	0.0478
Ether-linked phosphatidylcholine (PC O-42:9)	3	Database match (no MS/MS)	+2.90	0.0092

Metabolite Name	MSI Level	MS/MS Evidence	$\log_2$ Fold Change (BCAA vs. Placebo)	q-value (FDR)
Sphingomyelin (SM 8:1;2O/32:0)	3	Database match (no MS/MS)	+2.88	0.0169
Palmitoylethanolamide	3	Database match (no MS/MS)	+2.86	0.0119
Phosphatidylcholine (PC 34:3)	3	Database match (no MS/MS)	+2.80	0.0204
Ether-linked phosphatidylcholine (PC O-40:7)	3	Database match (no MS/MS)	+2.76	0.0232
Hypoxanthine	2	mzCloud MS/MS full match	−2.86	0.0119
Phosphatidylcholine (PC 36:3)	3	Database match (no MS/MS)	−2.80	0.0260
Lysophosphatidylcholine (LPC 20:1)	3	Database match (no MS/MS)	−2.76	0.0231
Lysophosphatidylcholine (LPC 17:2)	3	Database match (no MS/MS)	−2.72	0.0215
Sodium cholate	2	mzCloud MS/MS full match	−2.70	0.0184

Key metabolites highlighted in this study, including carnitine-related metabolites, fatty acid esters of hydroxy fatty acids (FAHFAs), and metabolites involved in the kynurenine pathway, as well as other representative significantly altered metabolites, are listed in this table. Metabolites were selected based on a fold change (FC) ≥ 1.2 or ≤ 0.83 and a Benjamini–Hochberg false discovery rate (FDR)-adjusted q-value < 0.1. Identification details, effect sizes ($\log_2$ fold change), and FDR-adjusted q-values are provided. These metabolites were significantly altered by BCAA supplementation compared with placebo in the post-exercise plasma metabolome.
